# Using 30-s Prone Back Extension Repetition Maximum Test to Predict Concentric and Eccentric 1 Repetition Maximum Squat Strength in Young and Older Adults

**DOI:** 10.1155/jare/6744171

**Published:** 2025-03-09

**Authors:** Michael T. Dunn, Phuong Quach, Monica McGraw, Robert C. Barefoot, Richard I. Preus, Donald H. Lein, Harshvardhan Singh

**Affiliations:** ^1^Department of Biology, University of Alabama at Birmingham, Birmingham, Alabama, USA; ^2^Department of Physical Therapy, University of Alabama at Birmingham, Birmingham, Alabama, USA; ^3^College of Medicine, University of South Alabama, Mobile, Alabama, USA; ^4^Human Performance and Nutrition Research Institute, Oklahoma State University, Stillwater, Oklahoma, USA

**Keywords:** aging, extension, loading, muscle, spine

## Abstract

**Background:** One repetition maximum (1RM) testing depends on lifting heavy loads which can put older adults at risk for injury and thus is nonfeasible. Thus, there is a great need for alternative 1RM testing methods, which are safe, patient-friendly, and clinically applicable, in older adults. Notably, aging-induced loss of muscle strength is greater for concentric than eccentric strength. However, there is a lack of information on unique 1RM for concentric and eccentric squat strength. Such information can lay the framework to design novel and effective resistive squat exercise programs in line with the principles of precision rehabilitation for various clinical populations.

**Purpose:** To investigate if the 30-s prone back extension repetition maximum test can predict 1RM concentric and eccentric squat strength in young and older individuals.

**Methods:** We enrolled and tested participants from 2 age groups: young: 21–35 years and older: 55–75 years in our cross-sectional study. Our main outcome measures were 30-s prone back extension repetition maximum and 1RM of concentric and eccentric back squat strength. All strength measures were normalized for body weight.

**Results:** Thirty-second prone back extension repetition maximum significantly predicted 1RM concentric (*p*=0.030, *ß* = 0.56; 95% CI: 0.006–0.102) and 1RM eccentric squat strength (*p*=0.041; *β* = 0.030, 95% CI: 0.001–0.058) in young and older adults, respectively. In addition, we obtained a trend toward significance for the relationship between 30-s prone back extension repetition maximum and 1RM eccentric (*p*=0.078) and 1RM concentric (*p*=0.066) squat strength in young and older adults, respectively.

**Discussion:** Novel data from our study show that 30-s prone back extension repetition maximum can predict 1RM of concentric and eccentric squat strength in young and older adults, respectively. Thus, clinicians and rehabilitation professionals can use our novel equations to design concentric- and eccentric-biased resistive training programs in young and older adults, respectively, without testing for 1RM.

## 1. Introduction

Progressive resistive squat exercise consists of lifting and lowering a load against a progressively increasing resistance in a squat position. This exercise is known to enhance the back extensor muscles, hip extensor muscles, and bones of the vertebral column [1]. We know that the one repetition maximum (1RM) assessment, which is the highest amount of load a tested muscle lifts and lowers through its full range of motion, is critical to design progressive resistive exercise programs [2–4]. Indeed, 1RM of the squat strength is feasible, reliable, and reported in both trained and untrained young adults [5, 6]. Nevertheless, high loading of the spine and hip may pose a risk while performing 1RM squat strength assessment, especially in clinical populations such as older adults with osteoporosis [7–9]. The high-loading nature of 1RM assessment can also be risky for young adults where an increasing incidence of osteopenia or osteoporosis is now being reported [10]. For example, ∼40% of young women, aged 16–35 years, had osteopenia at the spine in a previous study [11]. This is alarming because more than 50% of the fragility fractures occur in people with osteopenia [12]. Furthermore, the prevalence of osteoporosis can reach up to 50% in young adults with various chronic clinical conditions such as cystic fibrosis, secondary amenorrhea, Type 1 diabetes, thalassemia major, Crohn's disease, rheumatoid arthritis, and anorexia nervosa [13]. Thus, there is an urgent need to design novel ways, which are safe, clinically feasible, low cost, and user-friendly, to test the 1RM of the squat strength in young and older adults. Specifically, a safe way to assess 1RM of squat strength could help design clinically feasible and scientifically effective rehabilitation programs to strengthen the spine in young and older adults with musculoskeletal conditions.

We know that the 1RM concentric strength is the main determinant of conventional 1RM testing because the 1RM testing is dictated by the maximum amount of load that can be lifted. However, the muscular force produced via eccentric contractions is substantially greater than that produced via concentric contractions [14]. Thus, the eccentric phase remains suboptimally loaded for a typical 1RM assessment, resulting in less effective eccentric loading. This could be especially important for older adults. We know that the eccentric vs concentric strength of muscles is conserved to a greater degree with aging [15]. Further, the energy efficiency and lower cardiometabolic loading of eccentric vs concentric muscle contractions for a similar amount of muscle force strengthen the case for proper eccentric strengthening in older adults [16, 17]. Currently, there are no data on the unique 1RM of concentric and eccentric squats in young and older adults, which will be a critical requirement to uniquely maximize the concentric and eccentric strength of the squat exercise. In fact, such knowledge can provide the data required to generate systematically learned, novel, and effective rehabilitation programs for the enhancement of back extensor muscles and bones of the vertebral column.

A popular clinical test to assess back muscle strength is the 30-s prone back extension repetition maximum test [18]. The lifting and lowering of the upper thoracic region during the 30-s prone back extension exercise utilizes the concentric and eccentric muscle strength of the back extensors. Thus, the muscular action of the back extensor muscles during the 30-s prone back extension exercise mimics the squat exercise. Notably, the 30-s prone back extension exercise can safely increase the overall muscular strength of the back extensor muscles and reduce the risk of spinal injury in older adults or clinical populations [18, 19]. However, it is unknown if an individual's performance of the 30-s prone back extension exercise can predict the 1RM concentric and eccentric squat strength. A prediction equation derived from a safe, low-technology, and clinically relevant test to estimate 1RM concentric and eccentric squat strength can confer safety to planning and designing a high-intensity loaded squat resistive training program, especially in young and older adults with musculoskeletal conditions of the spine. Moreover, such information will be important to design progressive squat resistive concentric and eccentric squat exercise programs in situations where typical 1RM testing is not feasible due to fear of injury or lack of appropriate and safe equipment.

Thus, the main purpose of this study was to determine if the 30-s prone back extension repetition maximum test can predict 1RM concentric and eccentric squat strength in young and older adults. We hypothesized that 30-s prone back extension repetition maximum test will predict 1RM concentric and eccentric squat strength in young and older adults.

## 2. Methods

Our sample size is in line with a previous study design as ours [2]. Our power analysis yielded a sample size of 14 participants per group using a linear regression fixed model, single regression coefficient with an effect size (*f*^2^) of 0.7, alpha error probability at 0.05, and power at 0.8. Effect size of 0.7 ensured the robustness of our prediction equation. The inclusion criteria consisted of healthy independent ambulatory community-dwelling young (21–35 years) and older (55–75 years) adults. The exclusion criteria of our study are as follows: (1) uncontrolled diabetes; (2) uncontrolled hypertension; (3) any hardware in the body; (4) any back surgery/myocardial infarction/congestive heart failure/cataract surgery/stroke within previous 6 months; (5) known prior vertebral fracture; (6) known fragility fracture within the last year; and (7) back pain. In addition, we excluded any participants who reported tobacco use within the previous 10 years or current use of medications that affect muscle/bone, such as hormone replacement therapy or corticosteroids. Various agents such as tobacco or drugs such as hormone replacement therapy/corticosteroids affecting bone and muscle health could act as confounders. For example, tobacco [20] and corticosteroids [21] are well-established risk factors for low bone density and fracture, whereas the effect of hormone replacement therapy on muscle strength is debatable [22, 23]. The University of Alabama at Birmingham institutional review board approved this study.

### 2.1. Study Design

Our experiment was conducted using a cross-sectional design. Participants completed 2 visits, 3–7 days apart, in the Physical Therapy Research Laboratory. The main aim of the second visit was to assuage any learning effect from Visit 1 and obtain the main outcomes if not obtained during the first visit. Participants provided their informed consent during their first visit. Next, we performed their anthropometric measurements as detailed below. Then, based on a simple randomization technique, we determined the order of testing of the 1RM concentric and eccentric squat strength test, unique to each participant which was consistent across their 2 visits. Finally, we assessed the 30-s prone back extension repetition maximum test. The aforementioned order of testing was kept consistent for both the visits for each participant. The highest values obtained for the 30-s prone back extension repetition maximum test and 1RM concentric and eccentric squat strength test were used for all the data analysis. 1RM concentric squat strength and eccentric squat strength were normalized for body weight for all the reported analyses.

### 2.2. Anthropometric Measurements

Participants were asked to take their shoes off with nothing in their pockets for height and weight measurements. The height of the participants was measured in centimeters using a wall stadiometer (Novel Products Inc, Rockton, Illinois). We used a digital physician's scale (Tanita Corporation of America, Arlington Heights, Illinois) to measure the body weight (kg) of our participants. We calculated body mass index (BMI) using the formula, weight divided by height squared (kg/m^2^).

### 2.3. Warm-Up

All participants performed a warm-up session before the actual testing began. Participants used a stationary bike ergometer or an elliptical machine for 5 minutes at a relaxed pace for performing their warm-up. Next, we provided a rest interval of 90 s to the participants before any testing started.

### 2.4. Goniometer

A wireless digital goniometer (Biometrics Corp., UK) was secured on the lower back (L2–L5 spine) of the participant to record the number of repetitions for the 30-s prone back extension repetition maximum test after the participant rested in the prone position on a plinth. One complete wave, trough to trough, represented the completion of a single back extension repetition. In addition, a tester visually counted the total number of repetitions performed by the participant to tally with the goniometer recordings.

### 2.5. 1RM Concentric and Eccentric Squat Strength Tests

Individuals were asked to stand on a wooden platform placed beneath the Smith machine (Body-Solid, Forest Park, IL) and slowly descend to the required squat depth of 45-degree flexion angle of the knee (*n* = 29, knee flexion angle = 45.91 ± 3.09) with a wireless goniometer attached to their knee. Next, the corresponding position on the Smith machine was marked. We chose the position of partial squat (a 45-degree flexion angle of the knee) for all the squat tests because of its relative safety while maintaining its clinical utility for anabolic capability on the back extensors [24]. Moreover, the partial squat vs full deep squat can favorably stimulate the erector spinae muscle group [25]. To limit the risk of injury and to help in the maintenance of form, participants were instructed and guided into a proper squat form. There were trained spotters guarding the participant throughout the testing.

The 1RM concentric squat strength test consisted of finding the 1RM for the concentric squat, which involved raising the barbell. The barbell was first placed at the position described above. Next, participants were familiarized by performing 2–3 submaximal trials. Finally, the barbell was loaded, and participants would raise the barbell in a controlled manner until reaching an upright posture. Then, the participant locked the barbell in place. The testing team then returned the barbell to its original position between each trial. This was performed to prevent performing the eccentric phase of squat by the participants.

The 1RM eccentric squat strength test involved bringing the loaded barbell down from the participant's upright stand position to the standard 45-degree flexion angle of the knee position. The Smith machine barbell was secured in this eccentric starting position through hooks on the barbell that could lock into the holes of the frame of the Smith machine while safety locks for the barbell placed at 45-degree flexion angle of the knee position on the Smith machine prevented participants from going further down than the intention. The tester made sure that the Smith machine's barbell was fully rested on the participant's shoulder before the participant was instructed to bring the barbell down to the resting/safety stops in a controlled manner. To prevent participants from performing the concentric phase of squat, the research team raised the barbell and hooked it to the frame of the Smith machine at the starting position (that is, the participant's upright standing position).

Both the tests preceded with the instructions and conditions written above. The weight on the barbell was increased by ∼10–20% for each successive trial until a failure to perform concentric or eccentric phase was reached or until all 8 trials for the visit had been performed. The trial with the maximum value was used for all the analyses. A trial was deemed a failure if an individual could not complete the squat fully or there was a loss of form, such as a shaky movement. We also stopped the 1RM tests when the participants verbally conveyed their inability or unwillingness to continue with the 1RM tests.

### 2.6. 30-s Prone Back Extension Repetition Maximum Test

**Figure 1:**
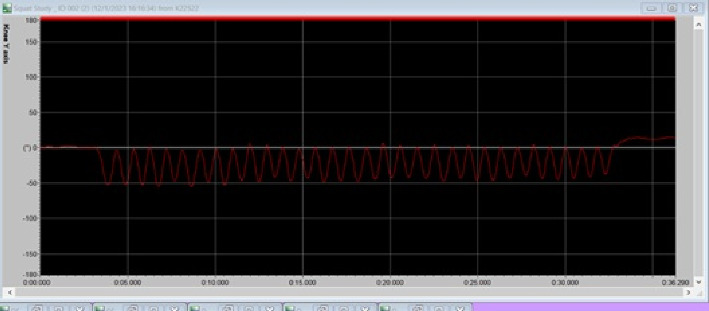
A representative example of the 30-s prone back extension test of a participant recorded via a wireless goniometer. The negative value of the trough represents extension movement.

The last test performed at the end of each visit for every participant was the 30-s prone back extension repetition maximum test. This consisted of two trials, with a rest interval of 90 s. The participants were tasked with performing as many back extensions as they could during a 30-s period. This test was performed with the participants lying prone on a plinth with their hands behind their head. The participants were aided by a research member who would stabilize their lower body by holding down their legs. All participants were asked to lift their chests off the plinth as high as they could and continue to lift their chest off the plinth for each repetition for the entirety of the 30-s prone back extension repetition maximum test. Importantly, all participants were familiarized before data collection. A consistent tester assessed and provided motivation to all the participants for all the tests. The same tester was responsible for observing the correct form of the 30-s prone back extension repetition maximum test via visual inspection. The wireless goniometer on the back was utilized to record the data for each 30-s prone back extension repetition maximum test trial. The goniometer was zeroed with each participant in the starting condition. During the trials, the goniometer would record and present a line with waves, with the trough of each wave representing the negative angle they reached upon raising their upper body as shown in Figure 1. A full back extension was defined as one trough to trough. A consistent tester counted the repetitions manually and matched their counts with the goniometer counts. The trial with the maximum value was used for all the analyses.

### 2.7. Statistical Analysis

We used the skewness, kurtosis, and Shapiro–Wilk tests to check for normality. The descriptive statistics of both the young and older adults are presented as mean ± standard deviation (SD) (Table 1). We also provide the descriptive data for men and women separately in Table 1. The absolute and normalized results for the 1RM concentric and eccentric squat strength and 30-s prone back extension repetition maximum tests are presented as mean ± SD in Table 1. Group differences for young vs older adults for the variables of 1RM concentric and eccentric squat strength and 30-s prone back extension repetition maximum tests were performed using independent sample *t* tests. All performance measures were normally distributed for men in both age groups, while all performance measures, except the 30-s prone back extension repetition maximum, were nonnormal for females. Thus, we used the Mann–Whitney test to report differences for all strength measures, except the 30-s prone back extension repetition maximum, in women only. Group-based (young vs. older) linear regression analyses were performed to analyze the relationships between the 30-s prone back extension repetition maximum test and the 1RM concentric and eccentric squat strength. The accepted alpha value was *p* < 0.05 in all statistical tests.

## 3. Results

Participants in our study were divided into two groups: young (21–35 years, *n* = 15; men = 9, women = 6; age = 23.67 ± 3.27 years; height = 1.69 ± 0.09 m; weight = 71.23 ± 13.4 kg) and older adults (55–75 years, *n* = 14; men = 8, women = 6; age = 58.64 ± 2.92 years; height = 1.74 ± 0.10 m; weight = 83.28 ± 12.72 kg).

Table 1 presents the values for both the absolute squat maximum and the normalized body weight values for all the strength and 30-s prone back extension repetition maximum tests. The absolute and normalized 1RM eccentric squat strength was lower by ∼24% and ∼34%, respectively, in older vs young adults (*p*=0.016 and 0.001, respectively). Similarly, the absolute and normalized 1RM concentric squat strength was lower by ∼30% and ∼39%, respectively, in older vs young adults (*p*=0.004 and *p* < 0.001, respectively). Further, we noticed that older men showed consistently lower values than young men for absolute (∼30.0%) and normalized 1RM eccentric (∼33.7%) (*p*=0.027 and 0.002, respectively) and concentric (*p*=0.006 (∼37.5%) and < 0.001 (∼39%), respectively) squat strength measures and 30-s prone back extension repetition maximum (*p*=0.038 (∼21.5%)).

**Figure 2:**
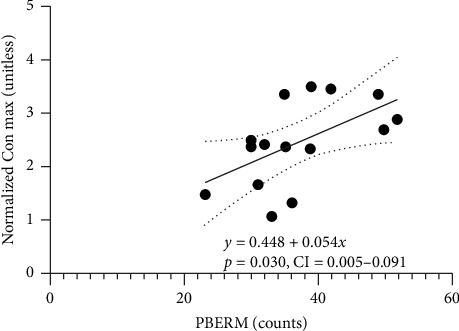
Linear regression of prone back extension repetition maximum (PBERM) vs normalized 1RM concentric squat strength (normalized Con. max) in young adults. 95% confidence interval is represented by the dotted lines.

**Figure 3:**
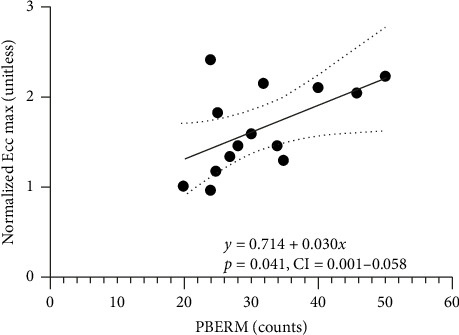
Linear regression of prone back extension repetition maximum (PBERM) vs normalized 1RM eccentric squat strength (normalized Ecc. max) in older adults. 95% confidence interval is represented by the dotted lines.

The novel significant regression equation predicting normalized concentric maximum based on 30-s prone back extension repetition maximum in young adults is shown in Figure 2. We also noted that the 30-s prone back extension repetition maximum trended to predict the normalized eccentric squat maximum (*p*=0.078) in young adults. The novel significant regression equation predicting normalized eccentric maximum based on 30-s prone back extension repetition maximum in older adults is shown in Figure 3. In older adults, the 30-s prone back extension repetition maximum trended to predict normalized 1RM concentric squat strength (*p*=0.066).

## 4. Discussion

To our knowledge, we are the first to report that the 30-s prone back extension repetition maximum test can significantly predict 1RM concentric squat strength in young adults and 1RM eccentric squat strength in older adults. Even the nonsignificant regression equations of 30-s prone back extension repetition maximum test predicting 1RM eccentric squat strength in young and 1RM concentric squat strength in older adults trended to be significant. Thus, our findings provide data showing vital clinical utility of 30-s prone back extension repetition maximum test in both young and older populations to safely predict their 1RM of concentric and/or eccentric squat strength. For example, various populations such as individuals with osteopenia, osteoporosis, rheumatoid arthritis, cystic fibrosis, or Crohn's disease, who display spine issues, may be contraindicated for lifting heavy loads for determining 1RM. Those populations can then safely perform a 30-s prone back extension repetition maximum test to determine the progressive dosage resistive exercise training regimen to strengthen the back extensors and the spine. Thus, healthcare practitioners can also feel confident when their patients, who typically might be contraindicated for 1RM testing, go for resistive exercise training programs to strengthen their back extensors and the spine. It should be noted, however, that clinical populations with back problems where the back extension movement is contraindicated such as people with spinal stenosis may not be a good fit for the 30-s prone back extension repetition maximum test.

Our results of overall and men-specific lower loss of eccentric than concentric squat strengths are in line with the well-established knowledge that aging induces greater loss of concentric than eccentric muscle strength [15]. Furthermore, our percentage loss of aging-induced eccentric and concentric squat strength is in line with previous literature reported for various other muscles such as the quadriceps femoris [26, 27]. Multiple factors can explain why 30-s prone back extension repetition maximum test predicted 1RM eccentric and 1RM concentric squat strength in older and young adults, respectively. The 30-s prone back extension repetition maximum test's predictive capabilities for 1RM concentric squat maximum in young adults could be dictated by the concentric-biased nature of the back extensor muscle contractions required for maximally performing the 30-s prone back extension exercise. The 30-s prone back extension exercise is performed through concentric contractions of the erector spinae muscle group, and this same muscle group is heavily activated during the partial squat used in the study [18]. In fact, both the back extension exercise and the concentric squat have been utilized to analyze the strength of and strengthening the back extensor muscles [18, 28]. Thus, the similar contraction of the same muscle group during both the concentric squat and 30-s prone back extensions, in part, explains the predictive ability of 30-s prone back extension repetition maximum test in young adults.

However, we did not find a similar result in older adults. The large significant drop in concentric strength, up to 50%, that occurs with age could contribute to the loss of this association between the two exercises [15]. Specifically, a large drop in concentric strength with age, in part, could explain why our older adults could not reach as high of a back extension range of movement as young adults. Further, lower strength of the back extensor muscles can contribute to thoracic kyphosis [10] which could put a biomechanical constraint on back extension performance. A known higher prevalence of thoracic kyphosis in older adults [29] could explain lack of association between concentric strength of the squat and 30-s prone back extension repetition maximum test in older adults. We also postulate that the time-constraint nature of the 30-s prone back extension repetition maximum test combined with the lower degree of back extension in older adults biased the performance of the test toward utilizing their eccentric strength, which is preserved to a greater degree with aging, of the back muscles. Utilizing the eccentric strength of the back muscles, older adults in our study could perform a quick controlled descent and thus perform maximally for the 30-s prone back extension repetition maximum test.

Sex-specific differences in squat strength and 30-s prone back extension repetition maximum test could also affect, in part, our findings. We noted that older men consistently displayed lower values of squat strength and 30-s prone back extension repetition maximum than young men. However, no such feature was noted for older vs young women. The physical activity status of our participants could dictate their performance on various tests utilized in this study. Although we did not collect observational data on the fitness of our participants, the seemingly enhanced fitness level of our older women participants may be a critical factor explaining lack of significant differences between young vs older women for squat strength measures and 30-s prone back extension repetition maximum. Our data show that there is potential for the development of sex-specific novel equations to predict 1RM concentric and eccentric squat strength from 30-s prone back extension repetition maximum test. Future studies could interrogate sex-specific regression equations for predicting 1RM concentric and eccentric squat strength based on 30-s prone back extension repetition maximum test performance.

### 4.1. Limitations

Our sample size and cross-sectional nature of the study limits the generalizability of our findings. Although our findings provide novel preliminary evidence to healthcare practitioners to start using the 30-s prone back extension repetition maximum to design resistive squat training programs in young and older adults, customized predictive equations may be required to account for the effect of sex or specific pathology of the participant. Although we noted sex-based differences in the performance indices of this study, our low sample size did not allow us to explore sex-based prediction equations. We know that sex is a critical variable affecting muscle degradation with aging [30]. Thus, future studies should examine if sex or specific pathology can affect the predictive ability of prone back extension repetition maximum test for 1RM concentric and eccentric squat strength in young and older adults. Not using free-weight squatting for squat testing in our study could also be deemed as another limitation. However, Smith machine has safety stops which can control the drop of barbell without putting strenuous effort on the participant. Thus, Smith machine allowed us to safely test 1RM squat strength test in our population.

## 5. Conclusions

Taken together, data from our study lay the framework for testing and validating the use of 30-s prone back extension repetition maximum test to predict 1M eccentric and concentric squat strength test in various populations. Our novel equations can potentially be used for constructing scientifically safe rehabilitation programs for strengthening the back extensor muscles and spine in young and older adults. Further, the 30-s prone back extension repetition maximum test is a low-cost, user-friendly, safe, low-technology, and clinically feasible technique. Thus, the translation of our findings to the real world is high. Future studies could use a larger sample to implement our findings to examine the feasibility of spine strengthening programs in young and older adults.

## Figures and Tables

**Table 1:** Characteristics of 1RM squat strength and prone back extension repetition maximum tests.

Variables	Participants (*n* = 29)		Two-sided P
	Young (*n* = 15) Male (*n* = 9) Female (*n* = 6)	Older (*n* = 14) Male (*n* = 8) Female (*n* = 6)	
1RM Ecc. (kg)	175.8 ± 53.6	133.8 ± 30.04	0.016

Male	184.71 ± 55.91	128.71 ± 33.98	0.027
Female	162.54 ± 51.85	140.61 ± 25.13	0.240
	Median = 167.83 min–max = 70.31–224.53	Median = 138.35 min–max = 102.06–170.10	

1RM Con. (kg)	171.80 ± 54.8	119.8 ± 26.80	0.004

Male	183.20 ± 54.97	114.53 ± 27.40	0.006
Female	154.60 ± 53.83	127.00 ± 26.72	0.240
	Median = 161.03 min–max = 74.84–224.53	Median = 136.08 min–max = 74.84–147.42	

Normalized 1RM Ecc.	2.494 ± 0.749	1.653 ± 0.477	0.001

Male	2.371 ± 0.641	1.410 ± 0.358	0.002
Female	2.679 ± 0.919	1.978 ± 0.436	0.180
	Median = 2.975 min–max = 1.23–3.48	Median = 2.099 min–max = 1.18–2.41	

Normalized 1RM Con.	2.447 ± 0.794	1.485 ± 0.451	< 0.001

Male	2.365 ± 0.698	1.245 ± 0.264	< 0.001
Female	2.569 ± 0.978	1.799 ± 0.475	0.240
	Median = 2.905 min–max = 1.31–3.48	Median = 1.940 min–max = 0.87–2.15	

Prone back extension repetition maximum	37.07 ± 8.242	31.43 ± 8.803	0.086

Male	37.89 ± 8.100	29.75 ± 6.341	0.038
Female	35.83 ± 9.065	33.67 ± 11.535	0.725

Abbreviations: 1RM, one repetition maximum; Con., concentric; Ecc., eccentric.

## Data Availability

The data that support the findings of this study are available from the corresponding author upon reasonable request.
